# Metagenomic analysis of healthy and diseased peri-implant microbiome under different periodontal conditions: a cross-sectional study

**DOI:** 10.1186/s12903-023-03442-9

**Published:** 2024-01-17

**Authors:** Liang Song, Ziying Feng, Qianrong Zhou, Xingwen Wu, Limin Zhang, Yang Sun, Ruixue Li, Huijuan Chen, Fei Yang, Youcheng Yu

**Affiliations:** 1 Department of Stomatology, Shanghai Fifth People’s Hospital, Fudan University, 801 Heqing Road, Shanghai, 200240 China; 2 Department of Stomatology, Zhongshan Hospital of Fudan University, 180 Fenglin Road, Shanghai, 200032 China

**Keywords:** Microbiota, Metagenomics, Dental Implant, Peri-implantitis, Periodontitis

## Abstract

**Background:**

Peri-implantitis is a polybacterial infection that can lead to the failure of dental implant rehabilitation. This study aimed to profile the microbiome of the peri-implant plaque and estimate the effect of periodontitis on it among 40 Chinese participants with dental implant prostheses and presenting with varying peri-implant and periodontal health states.

**Methods:**

Submucosal plaque samples were collected from four distinct clinical categories based on both their implant and periodontal health status at sampling point. Clinical examinations of dental implant and remaining teeth were carried out. Metagenomic analysis was then performed.

**Results:**

The microbiome of the peri-implantitis sites differed from that of healthy implant sites, both taxonomically and functionally. Moreover, the predominant species in peri-implantitis sites were slightly affected by the presence of periodontitis. *T. forsythia, P. gingivalis, T. denticola*, and *P. endodontalis* were consistently associated with peri-implantitis and inflammatory clinical parameters regardless of the presence of periodontitis. *Prevotella* spp. and *P. endodontalis* showed significant differences in the peri-implantitis cohorts under different periodontal conditions. The most distinguishing function between diseased and healthy implants is related to flagellar assembly, which plays an important role in epithelial cell invasion.

**Conclusions:**

The composition of the peri-implant microbiome varied in the diseased and healthy states of implants and is affected by individual periodontal conditions. Based on their correlations with clinical parameters, certain species are associated with disease and healthy implants. Flagellar assembly may play a vital role in the process of peri-implantitis.

**Supplementary Information:**

The online version contains supplementary material available at 10.1186/s12903-023-03442-9.

## Background

The application of dental implants to support fixed or removable prostheses is widely accepted as a treatment with high success rate and predictability [1, 2]. Technological innovations, including morphological design, surface modification, and special coatings, continue to improve the success rate of implant restoration [3–8]. However, several problems associated with the widespread use of implants have arisen over the years. Peri-implantitis is a significant cause of implant prosthesis failure [9–12]. Peri-implantitis is defined as an inflammatory reaction that affects both the soft and hard tissues surrounding the dental implant, which eventually results in loss of osseointegration [13, 14]. A history of periodontitis may be the main risk factor for the occurrence of peri-implantitis [15–19].

Similar to periodontitis, the occurrence of peri-implantitis is also related to bacterial biofilms [20, 21]. Regarding the composition of peri-implantitis-related biofilms, many key pathogens have so far been closely associated with peri-implant inflammation. However, there is still some controversy as to whether the submucosal biofilm composition around the implant is different from the one at the infected site of periodontitis, or even from around healthy dental implants [22]. Throughout the years, different techniques have been used to identify the microorganisms associated with peri-implantitis, including PCR-based assessment, hybridization, 16 S ribosomal RNA clonal analysis, and transcriptomic analysis [23]. However, comprehensive study of the subgingival microbiome around implants were incomplete until culture-independent techniques were widely adopted. Previous studies using conventional DNA probe and cultural analyses failed to discover the differences in the distribution of species between healthy and diseased implant sites [24, 25]. In currently available studies using culture-independent techniques, some taxa associated with periodontitis, such as *Porphyromonas gingivalis*, *Tannerella forsythia*, *Aggregatibacter actinomycetemcomitans*, *Treponema spp*. were described to be strongly related to peri-implantitis [26–29] and that the microbial composition of healthy and diseased implants differs [25, 26, 30–39]. The high-throughput DNA sequence analysis of 16 S rRNA is inadequate for the identification at the species level. Metagenomic sequencing makes up for this deficiency and provides further functionally relevant information [40–42]. The presence of residual periodontal pockets has been revealed as a risk factor for the infection around implants [15, 43]. While current studies of microorganism in submucosal plaque were more focused on differences between diseased and healthy implant plaque, instead of considering the influence of periodontitis on it.

In this work, we focused on the differences in the bacterial flora between peri-implantitis and healthy states and aimed to evaluate the effect of different periodontal health conditions on the peri-implant microbiota with the same health status of implants. By applying metagenomic sequencing to analyze the samples obtained from 40 Chinese participants who had received dental implant prostheses, we aimed to characterize the taxonomic composition and the functional features of the peri-implant microbiome. Differences in taxonomic and functional aspect were observed between the diseased and healthy implant sites, and their microbiota were affected by periodontal conditions.

## Methods

### Subject recruitment and sampling

The medical records of patients who had received dental implant surgery and visited the Department of Stomatology from January 2020 to February 2022, were reviewed. This study was composed of 40 randomly selected participants (18 men and 22 women of 50–80 years of age) who were systemically healthy and had at least one implant restored with crowns or prostheses for at least 1 year were included in this study. Participants were excluded if they were fully edentulous, had been using any medication known to affect periodontal health during the previous 2 weeks, had used systemic antibiotics in the past 3 months, were receiving prophylactic antibiotics or steroid medications or had a habit of heavy smoking (> 20 cigarettes/day). This study was conducted in accordance with the ethical standards of the institutional and/or national research committee and with the 1964 Helsinki Declaration and its later amendments or comparable ethical standards, and was approved by the Ethics Committee at Shanghai Fifth People’s Hospital affiliated with Fudan University [(2019) 101]. All participants enrolled in this study signed informed consent forms.

Before the survey, two authors received a standard consistency test of clinical parameter measurement. Clinical examinations of the sampling sites were performed independently by these two examiners. The kappa coefficient value greater than or equal to 0.90 is used, for which intra-examiner and inter-examiner reproducibility was determined, with high consistency. The following clinical parameters were recorded to evaluate the status of the peri-implant site: probing pocket depth (PPD) [44], radiographic peri-implant bone loss (RBL), bleeding on probing (BOP) scores [45], and the presence of suppuration (SUP). Clinical examinations were also performed on the remaining teeth to identify the periodontitis cohort with the following clinical parameters: PPD, BOP scores, and clinical attachment loss (CAL). The peri-implantitis lesions (peri-implantitis sites in oral cavity with established periodontitis [PD] and peri-implantitis sites in periodontally healthy oral cavity [PH]) were defined as PPD ≥ 6 mm and/or RBL ≥ 3 mm, with BOP and/or SUP [46]. The clinically healthy implant sites (clinically healthy peri-implant sites in oral cavity with established periodontitis [ND] and clinically healthy implant sites in periodontally healthy oral cavity [NH]) were restricted to PPD ≤ 4 mm and the absence of BOP, with no detectable evidence of radiographic bone loss [36]. The cohort identified as having established periodontitis (PD and ND) included participants who had been diagnosed with chronic periodontitis and had received periodontal treatment before implant surgery, and at enrollment examination, were detected to have more than two non-adjacent with BOP, PPD ≥ 4 mm, and CAL ≥ 3 mm present at the same time [47]. The cohort identified as having a clinically healthy periodontal condition (PH and NH) was based on the absence of BOP, PPD ≤ 3 mm, and no evidence of CAL in the remaining teeth at enrollment.

In total, we enrolled 40 patients for this study (PD:10, PH:10, ND:10, NH:10; 18 males, 22 females; mean age 65.8 ± 6.8 years), with one implant per patient. The study groups consisted of participants with a diseased implant and the presence of established periodontitis (PD, N = 10), a healthy implant with the presence of established periodontitis (ND, N = 10), and a diseased implant without periodontitis (PH, N = 10), a healthy implant without periodontitis (NH, N = 10) were compared for demographic and clinical features (Table 1).

**Table 1:** Demographic and clinical features of the study cohort

	PD(n = 10)	ND(n = 10)	PH(n = 10)	NH(n = 10)	*p*-value
Patient age (years ± SD)	64.3 ± 7.5	65.1 ± 6.7	69.4 ± 5.9	64.4 ± 2.1	0.28
Gender(M/F)	4/6	5/5	5/5	4/6	
Implant wear (years ± SD)	6.3 ± 2.6	5.8 ± 2.2	5.9 ± 2.2	6.4 ± 1.9	0.91
RBL (in mm, mean ± SD)	5.8 ± 1.2	0.2 ± 0.2	5.7 ± 1.4	0.2 ± 0.2	< 0.001*
PPD (in mm, mean ± SD)	7.2 ± 1.7	2.6 ± 0.8	7.0 ± 1.8	2.5 ± 0.8	< 0.001*
BOP proportion of sites per subject	100	0	100	0	< 0.001*
Suppuration proportion of sites per subject	30	0	20	0	< 0.001*

Note: PD: Peri-implantitis sites within periodontitis affected oral cavity; ND: clinically healthy implant sites within periodontitis affected oral cavity; PH: peri-implantitis sites within periodontally healthy oral cavity; NH: clinically healthy implant sites within periodontally healthy oral cavity. M: male; F: female. RBL: radiographic bone loss; PPD: pocket probing depth; BOP: bleeding on probing

* Statistically significant

In patients with peri-implantitis (PD and PH), submucosal biofilm samples were collected from the deepest PPD point in the sampling implant, whereas a random healthy peri-implant site (ND and NH) was selected for sampling in participants retaining successful implants. If more than one implant was assigned to the same clinical condition based on examination in the same patient, we randomly selected one of them as a sample site except those adjacent to a periodontitis site with PPD ≥ 6 mm. Before sampling, the implant sites were isolated using cotton rolls and air-dried. Sterile cotton pellets were used to remove the supramucosal biofilms. Submucosal samples were collected by inserting three sterile paper points (25#) into the base of the deepest probing depth and maintaining for 10 s. The samples were immediately placed in labeled Eppendorf tubes (Eppendorf, Hamburg, Germany) containing sterile PBS solution (Thermo Fisher Scientific, Waltham, MA, USA) and stored at − 80 °C for transportation to the laboratory for the subsequent extraction of DNA.

### Metagenome DNA extraction and Illumina shotgun sequencing

Total microbial genomic DNA samples were isolated using the E.Z.N.A Soil DNA kit (Omega Bio-tek, Norcross, GA, USA) (D5625-01), according to the manufacturer’s protocol. The isolated DNA was stored at -20 ° C. The prepared sample buffer was also subjected to laboratory-controlled extraction to identify any potential contaminants. The quantity and quality of the extracted DNAs were measured using a Nanodrop ND-1000 spectrophotometer (Thermo Fisher Scientific, Waltham, MA, USA) and agarose gel electrophoresis, respectively. The extracted microbial DNA was processed using the Illumina TruSeq Nano DNA LT Library Preparation Kit to construct metagenome shotgun sequencing libraries with insert sizes of 400 bp. Each library was sequenced using the Illumina NovaSeq platform (Illumina, USA) with PE150 strategy at Personal Biotechnology Co. Ltd. (Shanghai, China).

### Metagenomic analysis

The raw sequencing reads were processed to obtain quality-filtered reads for further analysis. Cutadapt was used to remove the sequencing adapters from the sequencing reads (v1.2.1) [48]. A sliding-window algorithm in fastp was adopted to trim the low-quality reads [49]. BMTagger was used to detect the reads aligned with the human host genome, and they were excluded to remove host contamination. After obtaining quality-filtered reads, taxonomic classifications of the metagenomic sequencing reads from each sample were conducted using Kraken2 [50] against a RefSeq-derived database, and their relative abundances within each sample were determined using QIIME [51]. Alpha diversities were calculated and presented using the Shannon index and Chao-1 index in the base R package [52], and beta diversities over taxonomic profiles were calculated based on the Bray-Curtis distance to describe the structural distribution of the samples through a two-dimensional ordination map in the Vegan R package [53]. Heatmaps were plotted using heatmap tools in a free online platform for data analysis named genescloud (https://www.genescloud.cn). This tool was developed from pheatmap package (V1.0.8) in R. The data was normalised by z-scores. The package uses popular clustering distances and methods implemented in dist and hclust functions in R. In this study we adopted the euclidean clustering distance and the complete clustering methods.

The core microbiome of the study groups was defined based on the taxa present with a mean relative abundance of ≥ 0.1% in each of the individual groups and a prevalence of ≥ 90% in all samples. Significant differences in the “core species” between the healthy implant and peri-implantitis groups with the same periodontal condition were determined by the Kruskal–Wallis rank sum test (*p* < 0.05) [34]. The effects of inflammation on the peri-implant microbiome under different periodontal conditions were examined using nonmetric multidimensional scaling (NMDS). The significance of the dissimilarity between the groups was evaluated using an analysis of similarity (ANOSIM) by applying the read abundance. A permutation test was performed in ANOSIM to provide a p-value, and the R-values were also calculated to reveal the statistical significance. The taxonomic profiles were compared using linear discriminant analysis combined with effect size analysis (LEfSe) in addition to Kruskal–Wallis and Wilcoxon tests to determine the differences and discover the potential biomarkers in the study groups based on the taxonomic abundance profiles [54]. The logarithmic linear discriminant analysis score was set to a threshold of 3.5 in the taxonomic analysis between peri-implantitis sites and healthy implant sites.

The functional profiles were acquired by annotating the Kyoto Encyclopedia of Genes and Genomes (KEGG) database [55]. Functional annotation of genes was performed with KOBAS (KEGG orthology-based annotation system) [56]. Principal coordinate analysis (PCoA) was used to characterize the differences in the functional profiles among the study groups. LEfSe analysis was performed to detect the differences in all functional groups based on the KEGG analysis. The logarithmic linear discriminant analysis score was set to a threshold of 3 in the functional analysis. Sequences from the metagenomic shotgun sequencing data were assigned to certain KEGG orthologous groups as well as to certain species based on the functional genes.

The correlations between the clinical parameters and the discriminating species in the peri-implant plaque were evaluated using Spearman’s rank correlation coefficient test by calculating the correlation coefficient value with the taxonomic relative abundances and clinical features in the periodontitis cohort (PD vs. ND) and periodontally healthy cohort (PH vs. NH). Those species with all correlation coefficients (R-value) less than 0.6 were not tested for statistical significance.

The demographic and clinical characteristics of the study population, including age, sex, implant wearing time, PPD, RBL, BOP, and SUP, were compared using the Kruskal–Wallis rank sum test. Statistical significance was set at *p* < 0.05. Benjamin and Hochberg’s false discovery rate was applied for multiple testing, and adjusted *p* < 0.05 was considered statistically significant.

## Results

### Clinical characteristics and summary of sequence

Forty partially edentulous Chinese participants with one dental implant per patient (18 males, 22 females; mean age 65.8 ± 6.8 years) were included in this study. There were no significant differences in age and gender, implant functioning time and implant location among the four groups. The details regarding the clinical examinations of each subject are available in Additional Table 1. Based on the analysis of clinical data from the sampled population, the healthy implant sites showed a lower PPD, BOP rate and peri-implant bone loss than the diseased sites. (Table 1).

The submucosal plaque samples were collected following the same validated and standardized protocol using sterilized paper points from the chosen implant sites in each subject. We subsequently performed whole genome shotgun sequencing of the plaque microbiome via Illumina NovaSeq to assess the role of the plaque microbiome in peri-implantitis and the effect of periodontitis on it. We noticed that the dominator in the microbiome we studied was bacteria, which is quantitatively over archaea; thus, we only performed the analysis on the bacterial microbiome.

As we mainly aimed to determine the differences in the microbiomes of peri-implantitis and clinically healthy implants, as well as the effect of a periodontitis condition on the peri-implant plaque, we only compared the implant sites with at least one similar environment, that is, we compared PD with ND and PH, and we also compared NH with PH and ND, but the differences between PD and NH will not be reported in the following description.

### Overall microbiome composition and biodiversity in peri-implant plaque based on metagenome

**Fig. 1:**
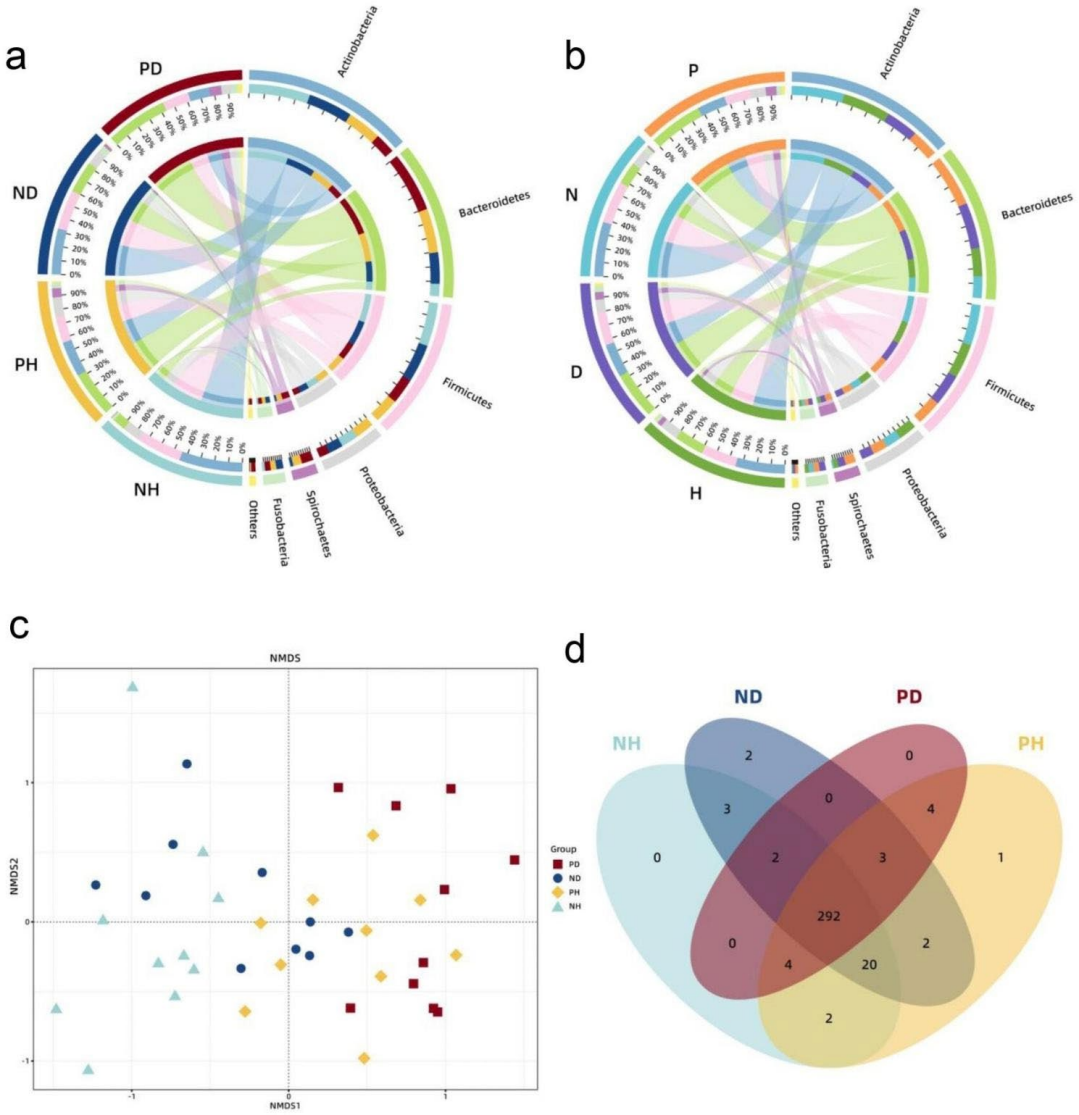
(**a**) The overall taxonomic composition of the four study groups and the correspondence between the taxa and sample groups at the phylum level shows different predominant taxa across groups. (**b**) The overall taxonomic composition of the four combined groups and the correspondence between the taxa and combined sample groups at the phylum level shows different predominant taxa across the combined groups. (**c**) NMDS plot of the four categories based on the Bray-Curtis distance indicates a clear distinction in clustering between the diseased and healthy implant sites. (**d**) Venn diagram shows that most species were detected in all four clinical categories

Eleven phyla were identified from all the samples collected. These were then further classified hierarchically into 20 classes, 33 orders, 53 families, 87 genera, and 335 species. A complete list of the species detected in all samples is shown in Additional Table 2. All 11 phyla were detected in each of the four clinically distinct groups. Over 96% of the total bacterial taxa identified within each group belonged to the top six phyla, composed of *Actinobacteria, Bacteroidetes, Firmicutes, Proteobacteria, Spirochaetes*, *Fusobacteria*, and their respective distributions are presented in a chord diagram (Fig. 1a).

The four different types of peri-implant niches had a reasonably different overall community composition at the phylum level. The differences in the relative abundance of Bacteroidetes and Spirochaetes between the groups were statistically significant (Kruskal–Wallis rank sum test, *p* < 0.05), with Spirochaetes being more abundant in both PD and PH than in ND and NH, and Bacteroidetes being more abundant in PH than in NH.

To determine whether the distributions of phyla presented remarkable differences between the healthy and diseased implant sites, and between study groups with or without periodontitis, the original grouping was combined into four new subgroups based on the health condition of peri-implant and periodontal tissue. The respective phyla between the four new subgroups were analogously analyzed and presented in another chord diagram (Fig. 1b). The overall compositions of the peri-implantitis subgroup and the periodontitis one were reasonably similar at phylum level. The microbiota of the peri-implantitis cohort (combined PD and PH sites; n = 20) was dominated by *Bacteroidetes* (37.7%), *Actinobacteria* (19.8%), *Firmicutes* (18.3%), *Proteobacteria* (10.6%), *Spirochaetes* (7.4%), and *Fusobacteria* (4.0%). Similarly, the cohort with periodontitis (combined PD and ND; n = 20) comprised *Bacteroidetes* (33.0%), *Actinobacteria* (23.4%), *Firmicutes* (23.0%), *Proteobacteria* (8.9%), *Spirochaetes* (5.2%), and *Fusobacteria* (4.5%). The microbiota from the cohort with healthy implants (combined ND + NH sites; *n* = 20) was dominated by *Actinobacteria* (38.1%), *Firmicutes* (29.4%), *Bacteroidetes* (15.8%), *Proteobacteria* (11.5%), *Fusobacteria* (3.5%), and *Spirochaetes* (1.2%). The cohort without periodontitis (combined PH and NH; n = 20) comprised *Actinobacteria* (34.5%), *Firmicutes* (24.7%), *Bacteroidetes* (20.5%), *Proteobacteria* (13.1%), *Spirochaetes* (3.4%), and *Fusobacteria* (3.0%). Qualitatively, the relative abundance of *Bacteroidetes* and *Spirochaetes* was significantly higher in the peri-implantitis sites (PD + PH sites) than that in clinically healthy peri-implant sites (ND + NH sites) (Kruskal–Wallis rank sum test, *p* < 0.05). In contrast, *Actinobacteria* were more abundant in the clinically healthy peri-implant sites than that in the peri-implantitis sites. In sites with established periodontitis (PD + ND), *Bacteroidetes* were significantly more abundant.

Non-parametric comparisons of the microbiota detected within each of the four clinical sites were processed with Kruskal–Wallis tests on the alpha diversity using.

the Chao1 index and Shannon index. Qualitatively, in the cohort with established periodontitis, submucosal plaque from the participants with peri-implantitis tended to have lower Chao1 richness and Shannon diversity index compared with the ones from participants who had clinically healthy implants, although neither of the differences reached statistical significance (Additional Fig. 1).

To further assess the differences in the composition of bacterial community between the samples, beta diversity analyses based on the weighted Bray-Curtis distance were computed, which considered the existence and abundance of the species in the community. NMDS analysis was performed with distance matrices and re-ordinations to intuitively present the distribution characteristics of the samples at the distance scale, and the results are shown in Fig. 1c. NMDS and ANOSIM revealed distinct microbiome profiles across the four groups. (R = 0.3227, *p* = 0.001).

### Bacterial associated with peri-implant health and disease in different periodontal conditions

Analysis of the quantitative taxonomic composition of the plaque microbiome via the QIIME2 pipeline highlighted a clear distinction between the microbiome of peri-implantitis and healthy implants, and also reflected the variations in the presence or absence of periodontitis.

**Fig. 2:**
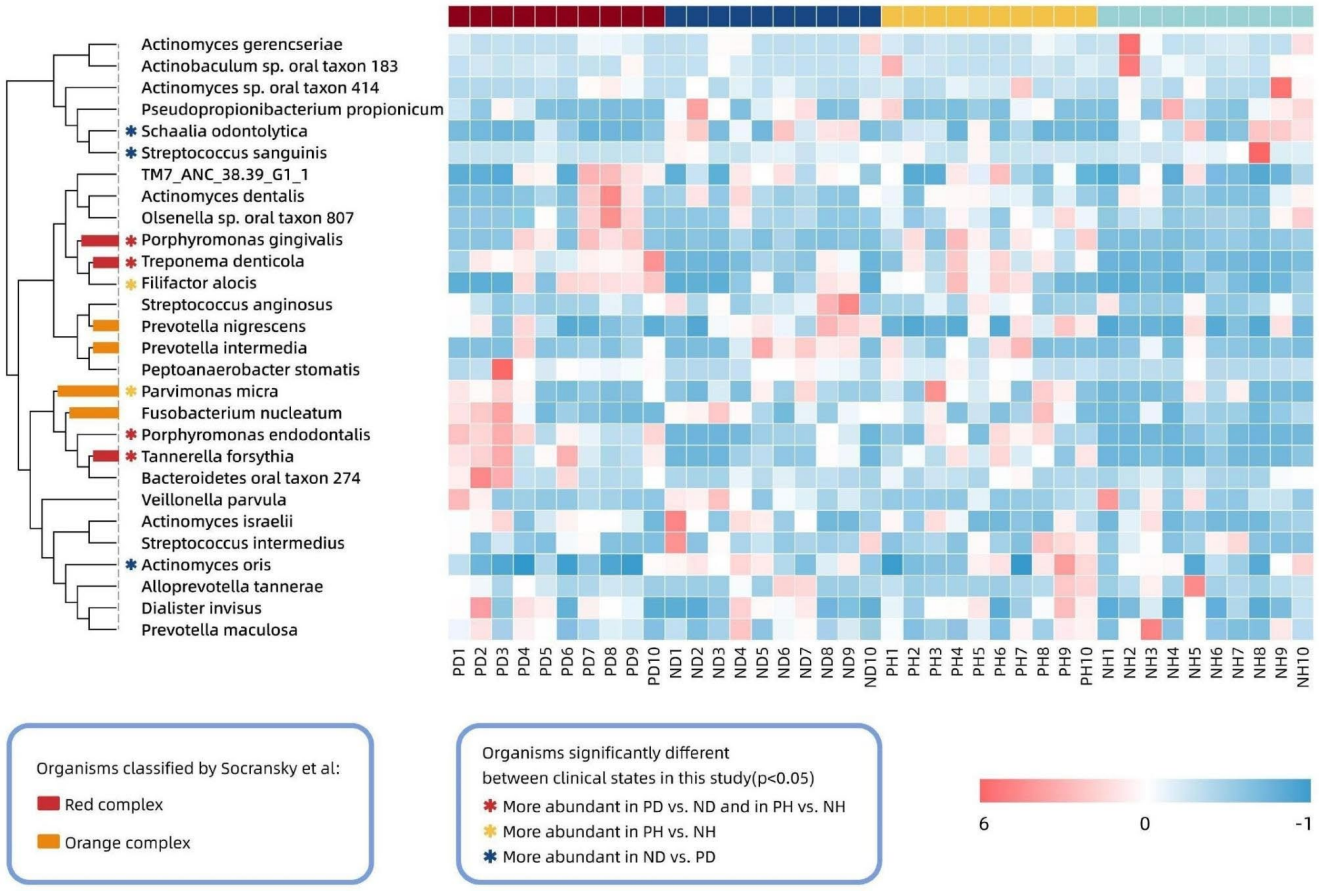
Heatmap of the 28 “core” species shows the distribution within the samples based on their relative abundance and normalized by the z-scores. The disease-associated (more abundant in the PD and PH groups) species and healthy (more abundant in ND group) ones are marked with asterisks on the basis of their significant differences. The members of the red and orange complexes are indicated with the branches in the clustering dendrogram on the left

Since the bacterial communities from the four clinical sites had similar levels of taxonomic diversity and 292 shared species (Fig. 1d), which accounted for 87.5% of the total species identified, we attempted to profile the “core species” that were present within the majority of samples. By setting the prevalence cutoff at 90% and the mean relative abundance within each group at > 0.1%, 28 “core species” were detected. The details of these species are described in Additional Table 3, and their distribution within the respective sites is represented in a heatmap (Fig. 2). In these “core species”, four were significantly more abundant (Kruskal–Wallis rank sum test, *p* < 0.05) in peri-implantitis sites despite the presence of periodontitis. These “peri-implantitis-associated” taxa were composed of the most widely acknowledged periodontopathogens: *Porphyromonas gingivalis*, *Tannerella forsythia*, *Treponema denticola*, commonly known as the “red complex,” and *Porphyromonas endodontalis*, another typical periodontal pathogen. Other significant differences were detected between the PH and NH groups in terms of *Filifactor alocis*, a newly putative periodontal pathogen, and *Parvimonas micra*, a member of the orange complex. They were also more abundant in PD than that in ND; however, the difference was not significant (Kruskal–Wallis rank sum test, *p* = 0.89 and *p* = 0.86, respectively). Some species were consistently more abundant in clinically healthy implant sites in the periodontitis environment, such as *Actinomyces oris*, *Streptococcus sanguinis*, and *Schaalia odontolytica*. Although they were also more abundant in NH than that in PH, the difference was not significant.

**Fig. 3:**
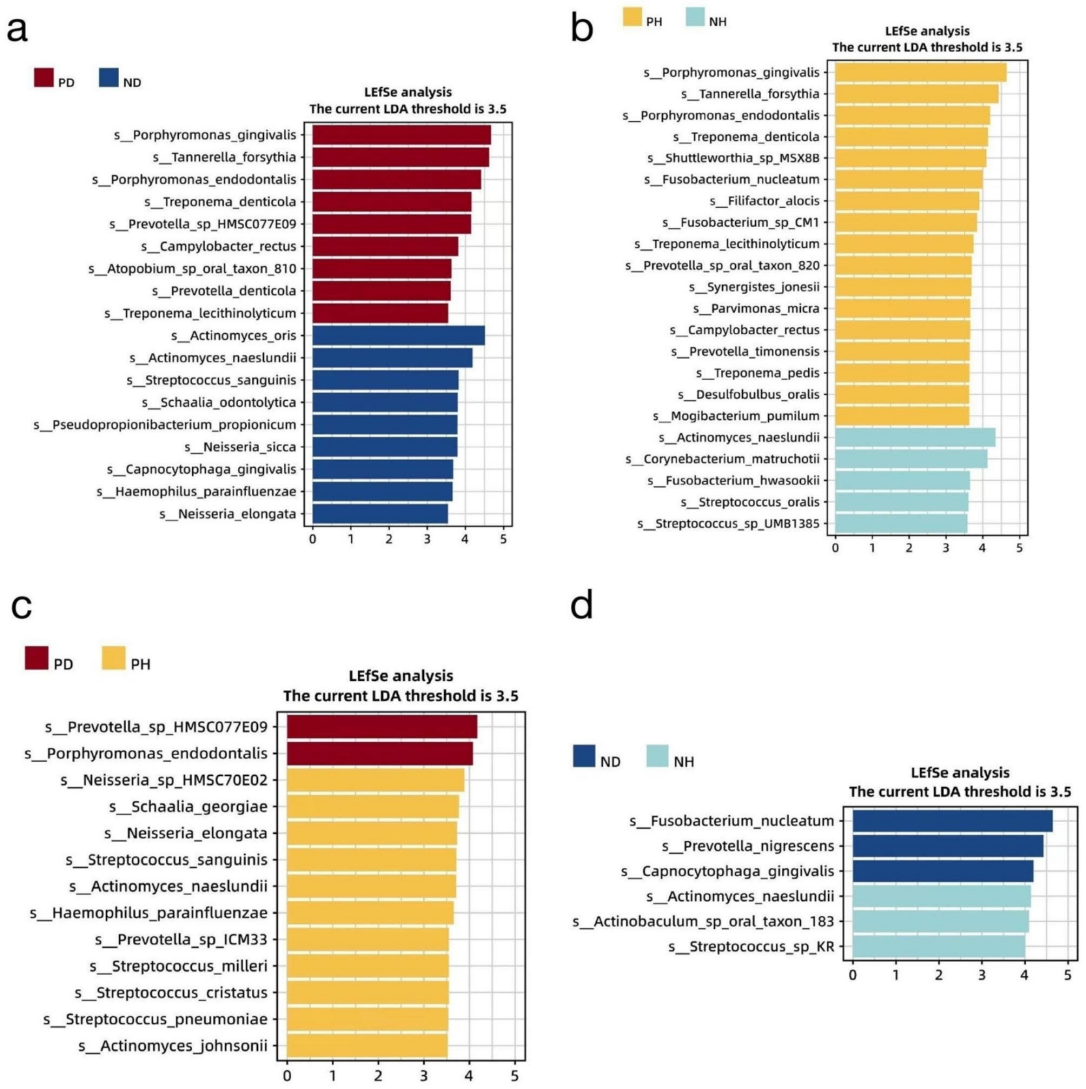
The LDA scores from the LEfSe analysis of the distinct species in the two specific groups: (**a**) PD vs. ND, (**b**) PH vs. NH, (**c**) PD vs. PH, and (**d**) ND vs. NH

LEfSe analysis was conducted to analyze the differential abundance across the four groups at the hierarchical taxonomic levels (Additional Table 4). At the species level, there was an overlap of “peri-implantitis-associated” taxa, between cohort with and without periodontitis, including *P. gingivalis*, *T. forsythia*, *P. endodontalis*, *T. denticola*, and *Campylobacter rectus* (Fig. 3a and b). Interestingly, an unclassified *Prevotella spp.*, namely *Prevotella sp.* HMSC077E09, along with *P. endodontalis*, was detected to have a significantly higher abundance in PD than that in both ND and PH (Fig. 3a and c), suggesting that these two species may be strongly related to peri-implantitis with the presence of periodontitis. *Actinomyces naeslundii* had a significantly high effect size in the healthy implant sites, and was more abundant even in NH over ND (Fig. 3d). *Actinomyces oris* was significantly more abundant in ND compared with PD, whereas *Corynebacterium matruchotii* was significantly more abundant in NH comparing with PH. In clinically healthy peri-implant plaque, the periodontitis pathogens *Fusobacterium nucleatum* and *Prevotella nigrescens*, both members of “the orange complex,” were significantly more abundant in ND compared with NH, indicating that the microbial community of a healthy peri-implant plaque may differ between the environment with the presence or the absence of periodontitis.

### Functional profile of microbiome in peri-implant plaque

**Fig. 4:**
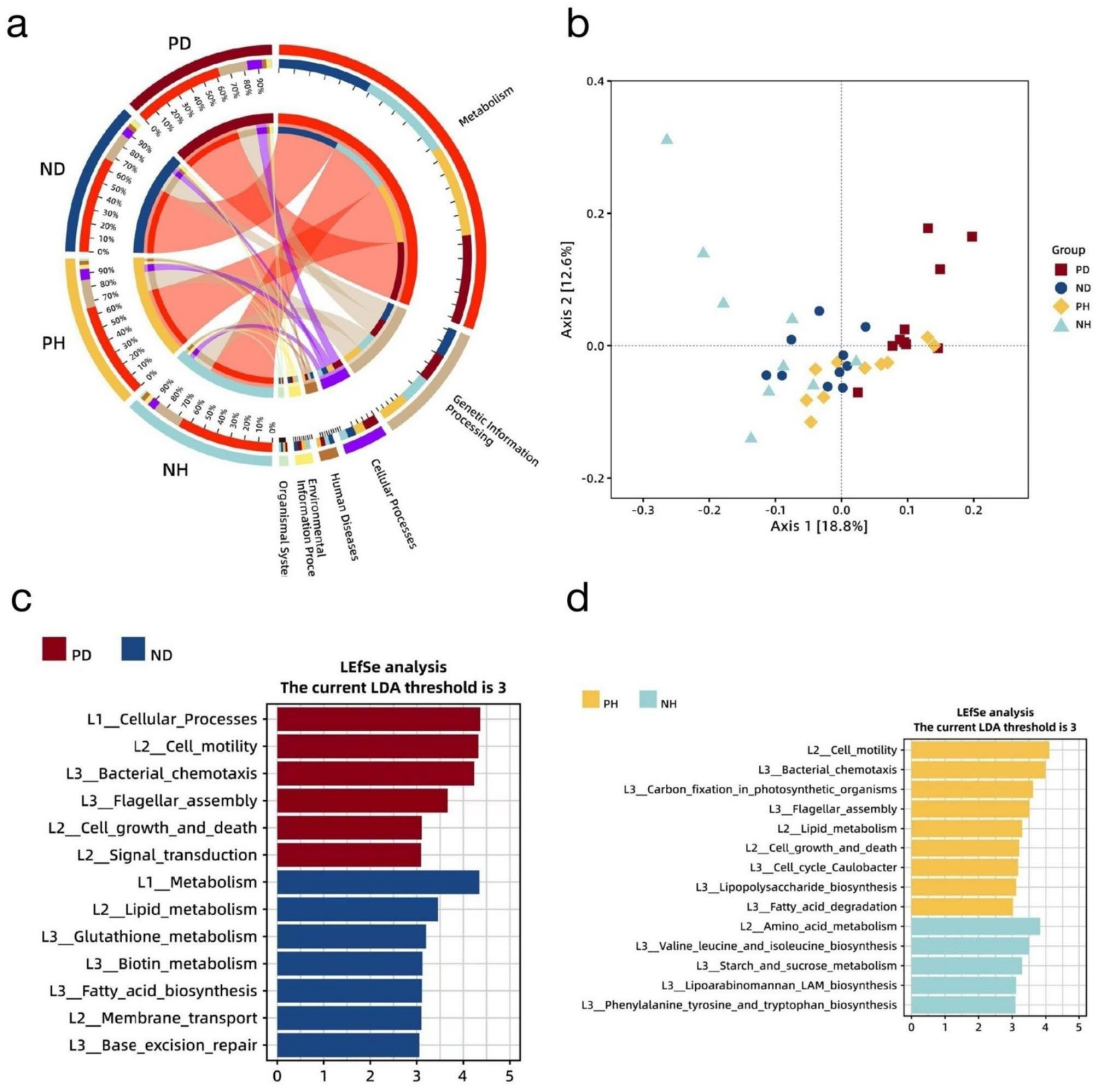
The Functional profile description of the four categories. (**a**) The overall functional composition based on KEGG of the four study groups and the correspondence between the functional unit and sample groups at KEGG Level 1. (**b**) PCoA plot of the four categories based on the Bray-Curtis distance indicates clear distinction across the groups. The LDA scores from the LEfSe analysis of the distinct function units between two specific groups, (**c**) PD vs. ND and (**d**) PH vs. NH

Dissimilarities in the functional composition across the distinct clinical statuses were revealed by examining the CDS profiles. The KEGG database was used in our functional analysis to analyze the metabolic pathways hierarchically [55]. At KEGG Level 1, “metabolism” was predominant in all samples, followed by “genetic information processing” (Fig. 4a). At KEGG Level 2, “carbohydrate metabolism” was predominant in all samples, followed by “amino acid metabolism” and “replication and repair” (Additional Fig. 2). The composition of KEGG Levels 1 and 2 was similar among the sample groups. However, the CDS profiles assigned by the KEGG database indicated that the functional composition of the four groups was distinct based on the PCoA plots (Fig. 4b), which was supported by ANOSIM (PD vs. ND: R = 0.198 and *p* = 0.001, PH vs. NH: R = 0.130 and *p* = 0.005). The LEfSe analysis revealed an overlap of differences between PD vs. ND and PH vs. NH in KEGG Level 3 (Additional Table 5), which included bacterial chemotaxis (ko02030) and flagellar assembly (ko02040) (Fig. 4c and d). Both were more abundant in the peri-implantitis sites despite the presence of periodontitis. Meanwhile, in the samples from participants without periodontitis, several other pathways were significantly more abundant in peri-implantitis sites, including carbon fixation in photosynthetic organisms (ko00710), cell cycle - Caulobacter (ko04112), lipopolysaccharide biosynthesis (ko00540), and fatty acid degradation (ko00071). This suggests that the presence of periodontitis may lead to a decrease in the advantageous variety in the functional structure of the peri-implant plaque.

**Fig. 5:**
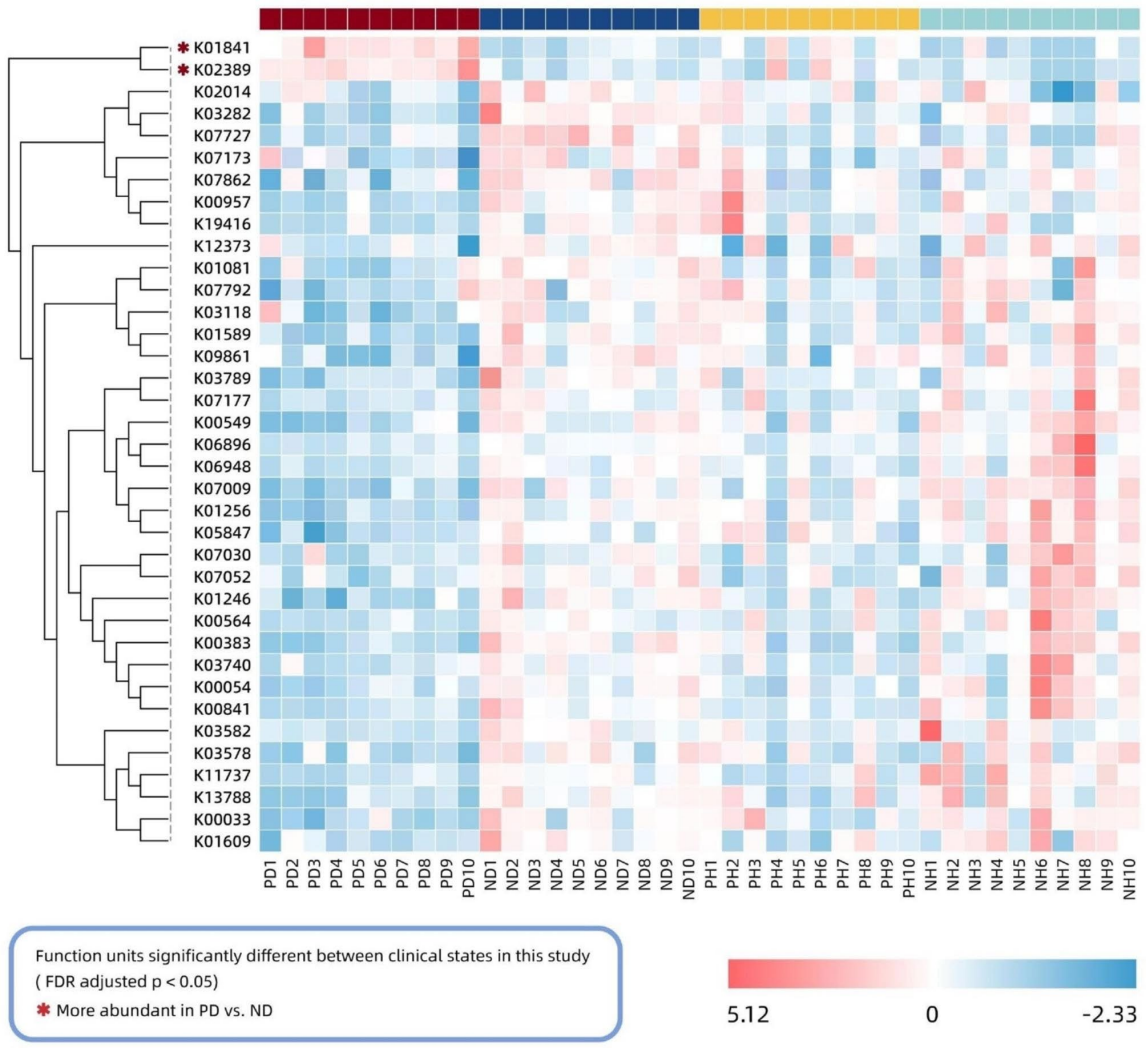
Heatmap of the 37 differential function units shows distribution within the samples based on their relative abundance. Peri-implantitis-associated function units are marked with asterisks

Kruskal–Wallis rank sum tests revealed differences in the read abundance of the CDS clusters of 37 function units in KEGG Level 4 between the peri-implantitis sites and the clinically healthy sites in the cohort with periodontitis (Kruskal–Wallis rank sum test with FDR-adjusted *p*-value < 0.05) (Fig. 5). No significant differences were observed in the read abundance of the CDS clusters of the two groups without periodontitis. Only two functional units were more abundant in the peri-implantitis sites in the periodontitis cohort, phosphoenolpyruvate phosphomutase (pepM, K01841), which belongs to the biosynthesis pathway of various antibiotics (ko00998), and flagellar basal-body rod modification protein (FlgD, K02389), which belongs to the flagellar assembly pathway (ko02040). We sought to determine the taxonomic origin of these two differential function units through the taxonomic assignment of the CDS clusters. Sixty-seven species were assigned (Additional Table 6), and 49.25% of them were species from the genera *Treponema, Selenomonas*, and *Campylobacter*, including some acknowledged and putative periodontal pathogens such as *Treponema denticola, Treponema socranskii, Treponema vincentii, Selenomonas noxia*, and *Campylobacter rectus*, suggesting that these three genera might be strongly relevant to the emergence of peri-implantitis in the cohort with periodontitis.

### Correlation of microbials species to the clinical parameter

**Fig. 6:**
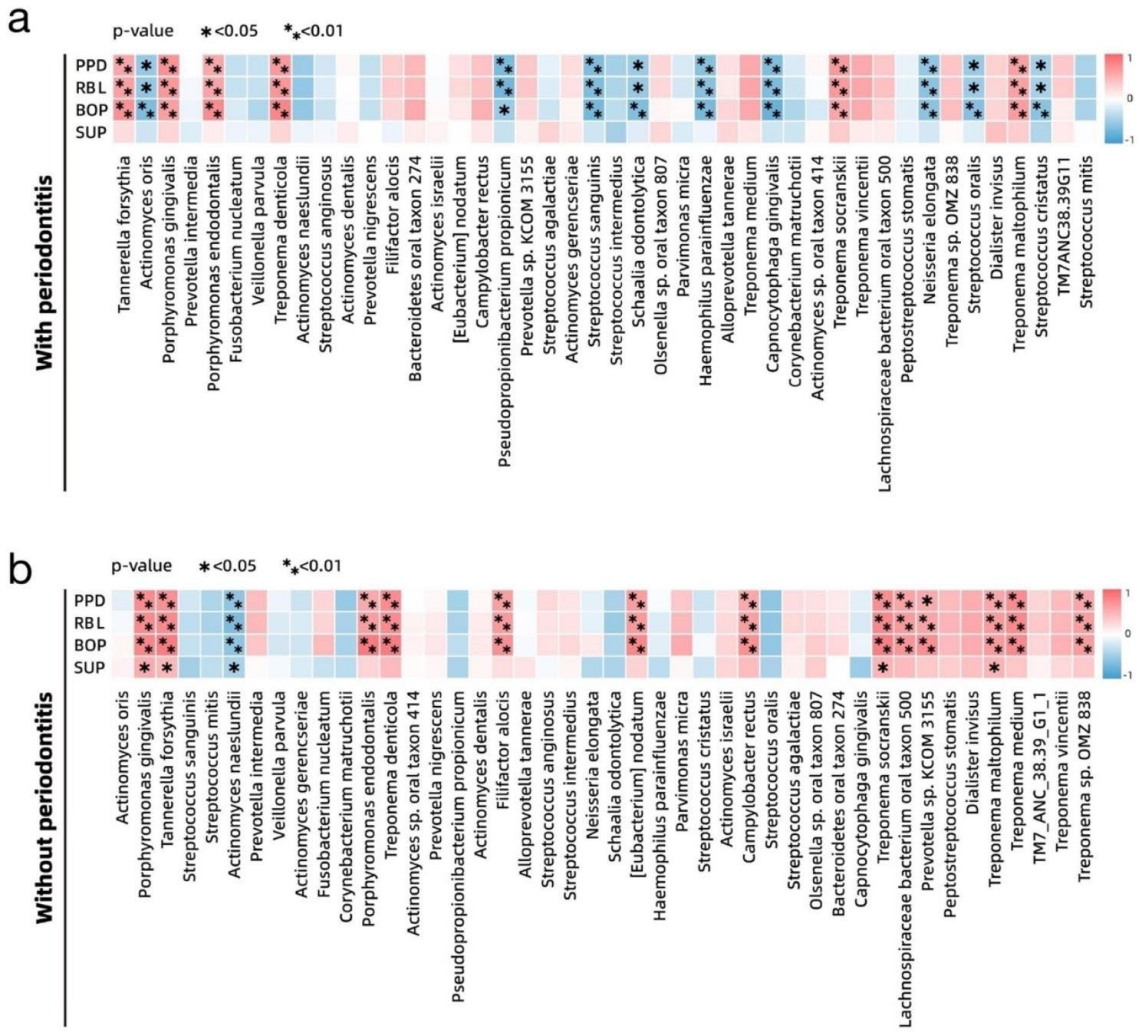
Correlation between the taxa and clinical parameters based on Spearman’s rank correlation coefficient test. In the cohorts with periodontitis (**a**) and without periodontitis (**b**). Heatmap of the 44 species with sufficiently high prevalence and abundance shows positive or negative correlations. The species showing significant correlation are marked with asterisks by their *p*-value

With the hypothesis that some species might be correlated with the clinical features of peri-implantitis, we performed Spearman’s correlation analysis to examine the correlation between the species and clinical parameters in the subject group. To exclude species that are rare and with low abundance so that significant correlations may be demonstrated, we set the prevalence cutoff in all samples at 50% and the overall abundance cutoff at 0.01%. The details of the results are shown in Additional Table 7. The four typical disease-associated species, *P. gingivalis, T. forsythia, T. denticola, and P. endodontalis*, along with three other *Treponema* species (*T. socranskii, T. medium*, and *T. maltophilum*), were significantly positively correlated with the clinical parameters of peri-implantitis (PPD, RBL, and BOP), despite the presence of periodontitis (Fig. 3). Regarding the species negatively correlated with the clinical parameters, some aerobic/facultative bacteria were detected, including species from the phylum *Actinobacteria*, such as *A. oris, Pseudopropionibacterium propionicum*, and *Schaalia odontolytica* along with *Streptococcus sanguinis* and *Streptococcus oralis* in the cohort affected by periodontitis (Fig. 6a). In contrast, only *A. naeslundii* was significantly negatively correlated with these parameters in the periodontally healthy cohort. Conversely, more anaerobic bacteria, such as *Filifactor alocis* and *Campylobacter rectus*, were positively associated with the clinical parameters of peri-implantitis in the periodontally healthy groups than that in those with periodontitis (Fig. 6b).

## Discussion

In this study, we systematically compared the taxonomic and functional composition of the submucosal plaque microbiome of clinically healthy and diseased peri-implant sites within a group of individuals with or without established periodontitis via metagenomic analysis to characterize the microbiome of peri-implantitis and to assess the effect of periodontitis on the microbiome of the submucosal plaque. We observed evident differences in both taxonomic and functional composition of the submucosal microbiome associated with distinct health conditions of dental implants. Here, the core microbiome of peri-implant plaque was delineated, which is considerably in accordance with previous findings in other high-throughput sequencing studies [34, 35, 38, 39]. This suggests that these species constitute a fundamental structure of the peri-implant submucosal microbiofilm.

It has been speculated that peri-implantitis is also a biofilm-induced infection, such as periodontitis, and the etiology of peri-implant inflammation should be associated with multi-bacterial interactions within the biofilm community, instead of one exact pathogen. In 1998, Socransky et al. proposed the “complex theory” based on certain associations between the severity of periodontitis and some particular species [57]. Our data also revealed that a group of specific species in the microbial community of the peri-implant plaque, with their remarkably high relative abundance, and their correlation with clinical parameters, may be positively associated with the disease state of the implants [26–29, 35, 58, 59]. In general, the predominant species of the peri-implantitis microbiome usually consists of aerobic gram-negative bacilli, facultative anaerobic, and anaerobic species [60]. Interestingly, some of the species suspectedly associated with peri-implantitis shared considerable overlap with classic periodontal pathogens, including those described in this study: the red complex (*P. gingivalis*, *T. forsythia*, and *T. denticola*) and *F. nucleatum* from the orange complex [28, 35, 36, 58, 59, 61]. Results from previous observations have validated that pathogenic taxa from periodontitis may be shared between teeth and implants [62–64]. New putative periodontal pathogenic taxa such as *P. endodontalis, Filifactor alocis*, and *Parvimonas micra* [65, 66] were observed to have increased abundance and prevalence in the peri-implantitis microbiome, as demonstrated in other observations [22, 27, 35, 36, 58, 67]. Correlations between particular taxa present in peri-implantitis lesions and the disease clinical parameters corroborate the speculation. As *P. gingivalis* and *F. alocis* were observably more abundant in sites with severe periodontitis and deeper pocket depths compared with healthy periodontal sites [68], we found that they were both positively correlated with PPD, RBL, BOP, and SUP in peri-implant sites. In addition, *Treponema* spp. was positively associated with the clinical parameters in previous studies on peri-implantitis [28, 34], which was identified at the species level, namely, *T. socranskii, T. medium*, and *T. maltophilum* in this study. They were revealed to be relevant to the inflammatory state of the dental implant [35, 38, 58, 59]. Notably, the peri-implantitis-associated species mentioned above were significantly more abundant in diseased implant sites even in the cohort without periodontitis, implying their prosperity would have a profound and steady influence on the emergence and progression of the peri-implantitis [35, 36, 69].

We also noted a higher incidence of *P. endodontalis* in sites with periodontal disease than in periodontally healthy sites. *P. endodontalis* was identified as a novel putative periodontal pathogen [67, 70] and has been reported to cause osteoclastogenesis [71]. The pathogenesis of periodontal disease associated with *P. endodontalis* may be dependent on NOD2 as it has the highest NOD2 stimulatory activity in a study conducted by Marchesan et al. [72]. The genus *Prevotella* has been frequently associated with peri-implantitis in previous studies [26, 28, 34, 58, 59, 73]; although the genus *Prevotella* did not show a significantly higher abundance in peri-implantitis than that in healthy implant sites in the current study, one of its unclassified species, *Prevotella* sp. HMSC077E09, together with *P. endodontalis*, was remarkably abundant in the peri-implantitis sites with established periodontitis, compared with the healthy implant sites in the periodontitis cohort and the peri-implantitis sites in the unaffected periodontal environment in our study group. Therefore, our results may provide a novel insight regarding the special role it plays in the disease process of peri-implantitis with the presence of periodontitis.

Concerning species associated with the clinically healthy condition of a dental implant, previous studies have presumed that particular taxa from genera *Actinomyces*, *Corynebacterium*, *Rothia, Streptococcus*, *Neisseria*, and *Kingella* [22, 34–36, 38, 73] may play constructive roles in preventing dysbiotic states of the implant plaque; we similarly identified species belonging to genera *Actinomyces*, *Corynebacterium*, and *Streptococcus*, for example, *A. naeslundii, C. matruchotii*, and *Streptococcus sanguinis*. These facultative anaerobic gram-positive bacteria are also usually related to the periodontally healthy condition [74–76],which aligned with the results of previous studies on clinically healthy implants [34–36, 73, 77, 78]. *Schaalia odontolytica* and *Pseudopropionibacterium propionicum* were the other species associated with healthy peri-implant sites and clinical parameters in the present study. Interestingly, they were both potential hosts of *Candidate Phylum Saccharibacteria* [79, 80]. *Saccharibacteria* (TM7) survives as an obligate epibiotic symbiosis on the surface of their host. It is considered a putative pathogen that is strongly associated with dysbiotic microbiota [28, 34, 74] and is positively correlated with inflammatory parameters [73]. However, one of the latest findings in mice reported that the TM7 species remodulated and downregulated host bacterial pathogenicity, thereby reducing inflammation and bone loss, which would be a protection to the periodontal tissues [81]. Further details regarding this symbiotic taxon and its biological regulation mechanism are necessary to understand the specific role it plays in microbial communities.

Here, we observed evidently higher level of both *F. nucleatum* and *P. nigrescens* in unaffected peri-implant plaque from subjects with periodontitis, comparing to periodontally healthy subjects. As putative pathogens may be present in healthy periodontal sites, but at lower levels than in diseased sites, these taxa were reported to function as a “bridging species” to link early colonizers with later ones [82]. A recently published paper showed that *F. nucleatum* colonization occurs at the stage of peri-implant mucositis, which is prior to the occurrence of peri-implantitis [36]. Another study reported no significant difference in the relative abundance of *F. nucleatum* between periodontal disease and healthy status and speculated that it may play a structural role in the microbial community [74]. The subgingival microbiota in patients with periodontitis can also survive in the peri-implant submucosal communities [78]. It has been reported that submucosal microbiofilms from healthy implants tend to harbor a higher number of pathogenic taxa in periodontitis subjects [83]. Other observations have implied that submucosal plaque harboring putative pathogens does not necessarily lead to peri-implantitis [16, 84], even though the presence of periodontal disease is one of the two known risk factors for peri-implantitis [18, 85]. It has also been proposed that more than a few clinically healthy peri-implant sulci present a microbial community structure that is “pre-dysbiotic”, where the number of health-associated taxa is not necessarily reduced, but the proportion of disease-associated taxa may gradually increase. This may develop into a disease state of peri-implant tissues [86].

Our results on functional assignment revealed that the microbial functional profiles of the peri-implantitis sites were distinct from that of those healthy sites. An analysis based on the KEGG database indicated that two typical functional units were enriched in peri-implantitis sites in the periodontitis cohort. These included genes encoding flagellar basal-body rod modification protein (FlgD) and phosphoenolpyruvate phosphomutase (pepM). These findings suggest that these two functional units may play a special role in the occurrence and development of peri-implant diseases. Studies have shown that FlgD is closely related to the invasion of epithelial cells as its expression is related to flagella, which is required in host cell invasion [87]. FlgD is a scaffolding protein needed for the pathway of flagellar assembly [88]. There is a link between it and the pathway of bacterial chemotaxis, the activity of which is reported to be upregulated in participants with chronic periodontitis [35, 89] and they were both considered as virulence factors in periodontitis [90, 91]. It has also been reported that the genes encoding flagellar assembly and bacterial chemotaxis-related proteins are enriched in periodontitis [92]. Functional analysis using KEGG in Shiba’s study showed that fliC, a function unit from flagellar assembly pathway, was one of the three functional units that are most abundant in both peri-implantitis and periodontitis [58]. All these findings support the notion that the function of flagellar assembly plays a vital role in the process of peri-implant disease. However, little is known about the role of pepM in the progression of peri-implant inflammation. Hidaka et al. [93] reported that some C-P compound-producing actinomycetes exhibit pepM activity and that pepM catalyzes the first C-P bond formation in the bialaphos biosynthetic pathway. Actinomycetes are commonly associated with a healthy periodontal or peri-implant state. Therefore, the effect of pepM and the C-P bond on the development of the disease needs to be further examined.

Since the quantitatively dominant species are not always the functionally dominant species [58], we also assigned the species with typical function to peri-implantitis sites. According to the assignments made with the KEGG database, the two distinguishing functional units highly abundant in peri-implantitis sites with periodontitis were mainly derived from flagellated motile species, including *Treponema, Selenomonas*, and *Campylobacter*, which is consistent with previous observations that these species were associated with periodontitis [31, 74, 94]. Research has also shown that *Treponema vincentii, Selenomonas noxia*, and *Campylobacter concisus* were only observed in sites with peri-implantitis but not periodontitis [95, 96]. However, these species were not identified as the most taxonomically abundant species in this study, emphasizing that these flagella-related gene-expressing bacteria may be essential to the pathogenesis of peri-implant diseases. In contrast, the “keystone pathogen” hypothesis highlighted that the potential importance of the low-abundance species should not be underrated and that particular low-abundance pathogen taxa can transform the bacterial community structure and behavior characteristics and may serve as an indicator of the microbiome shift from symbiosis to dysbiosis, as well as a potential biomarker of pathogenesis [97].

The results and conclusions drawn from this study should be considered cautiously as only a limited number of participants were recruited in the present metagenomic analysis (n = 40), which affects the generalizability of results. The widely adopted standardized protocol of sampling may have restricted the ability to harvest a completely comprehensive microbial biofilm with paper points from the peri-implant sulcus. There may exist some species that had been underestimated but are crucial in the disease process of peri-implantitis; thus, further studies with larger sample sizes are necessary to assess the pathogenic potential of these disease-associated species and their particular effect on peri-implant biofilms during the course of peri-implantitis. It’s worth noticing that there exist many factors that contribute to confounding in results of culture-independent microbiome studies throughout the process, from determining the homogeneity of the subject population, to the sample collection, handling and preservation of biological specimens, and to evolving approaches in laboratory process with elevated potential for batch effects. Therefore, it’s advisable to be more cautious at experiment design and results interpretation. Participant with different oral hygiene conditions, socioeconomic status and genetic predispositions may also lead to differences in results. Host-associated microbial communities are influenced by both host genetics and environmental factors. It has been reported that 5–45% of inter-individual variation can be explained by genetics [98], and oral microbiome is heritable, indicating by a large twin oral microbiome study [99]. The genetic variant may alter the microbiome directly, which can result in the disease phenotype. Although twin pair analysis on peri-implant submucosal plaque to explore the influence of genetic predisposition has not been conducted, we should not neglect its potential effect in the development of peri-implant inflammation. Batch effect is the systematic, non-biological differences between batches, it’s ubiquitous in genomics experiment and may mislead the conclusion. To minimize the impact of it, sound experiment designs and statistical analysis methods are necessary. A software named OSAT is designed to assign collected samples across batches in an appropriate way to handle batch effects [100].Future study should take sample-to-batch allocation into concern to reduce the confounding or correlation between batches and the biological variables of interest. Apart from this, contamination involving high-throughput sequencing can originate from environmental sources, such as extraction kits, plastic consumables and reagents, and also cross-contamination from other samples, which is beyond the control of the researchers, and can bias results of metagenomic studies [101, 102]. A standard checklist and strict decontamination protocols are suggested to prevent contamination, and contaminated data can be cleaned up with certain tools [103–105]. Future high-throughput sequencing experiment should also address the bias from contaminations to their conclusions. As the design of this study is set to be cross-sectional, data collection was performed at a single point in different individuals, which makes it inadequate to provide account for long-term implant health consequences compared to longitudinal study design with follow-up. Further studies on long-term dental implant outcome are preferable to be designed as longitudinal.

## Conclusion

The data from the present study indicated that the submucosal microbial compositions of peri-implantitis and healthy implant sites are distinct and are affected by the periodontal environment in which the implant is located. *P. endodontalis* and *Prevotella* sp. HMSC077E09 were found to be more abundant in periodontitis-affected peri-implantitis sites than in periodontally healthy ones. Red complex species and *P. endodontalis* were consistently associated with peri-implantitis regardless of periodontal condition, and were affirmed by their positive correlation with diseased clinical parameters. Conversely, in periodontitis affected cohort, *A. oris, S. sanguinis, P. propionicum* and *S. odontolytica* were associated with successful implant and aligned with their negative correlation with diseased clinical parameters. On the other hand, in the subjects unaffected by periodontitis, *A. naeslundii* was associated with clinically healthy implant by its taxonomic abundance and the negative correlation with clinical features. Function relevant to epithelial cell invasion, such as flagellar assembly, was detected to be enriched at peri-implantitis sites. This was interrelated to species from *Treponema, Selenomonas*, and *Campylobacter* genus that were not taxonomically thriving but may act as a fundamental trigger in the generation of peri-implant disease.

### Electronic supplementary material

Below is the link to the electronic supplementary material.

Supplementary Material 1

Supplementary Material 2

## Data Availability

The data used and analyzed during the current study are available from the corresponding author upon reasonable request.

## Abbreviations

PD: Peri-implantitis sites within periodontitis affected oral cavity
ND: clinically healthy implant sites within periodontitis affected oral cavity
PH: peri-implantitis sites within periodontally healthy oral cavity
NH: clinically healthy implant sites within periodontally healthy oral cavity
M: male
F: female
RBL: radiographic bone loss
PPD: pocket probing depth
BOP: bleeding on probing

## References

[1] Aglietta M, Siciliano VI, Zwahlen M, Brägger U, Pjetursson BE, Lang NP (2009). A systematic review of the survival and complication rates of implant supported fixed dental prostheses with cantilever extensions after an observation period of at least 5 years. Clin Oral Implants Res.

[2] Jung RE, Pjetursson BE, Glauser R, Zembic A, Zwahlen M, Lang NP (2008). A systematic review of the 5-year survival and complication rates of implant-supported single crowns. Clin Oral Implants Res.

[3] Schünemann FH, Galárraga-Vinueza ME, Magini R, Fredel M, Silva F, Souza JCM (2019). Zirconia surface modifications for implant dentistry. Mater Sci Eng C Mater Biol Appl.

[4] Peixoto CD, Almas K (2016). The implant surface characteristics and peri-implantitis. An evidence-based update. Odontostomatol Trop.

[5] Carinci F, Lauritano D, Bignozzi CA, Pazzi D, Candotto V, de Santos P et al. A New Strategy against Peri-Implantitis: Antibacterial Internal Coating. Int J Mol Sci. 2019;20(16). 10.3390/ijms20163897 PMC6720572 31405061

[6] Oyonarte R, Pilliar RM, Deporter D, Woodside DG (2005). Peri-implant bone response to orthodontic loading: part 1. A histomorphometric study of the effects of implant surface design. Am J Orthod Dentofacial Orthop.

[7] Rossi S, Tirri T, Paldan H, Kuntsi-Vaattovaara H, Tulamo R, Närhi T (2008). Peri-implant tissue response to TiO2 surface modified implants. Clin Oral Implants Res.

[8] Moraschini V, Poubel LA, Ferreira VF, Barboza Edos S (2015). Evaluation of survival and success rates of dental implants reported in longitudinal studies with a follow-up period of at least 10 years: a systematic review. Int J Oral Maxillofac Surg.

[9] Jung RE, Zembic A, Pjetursson BE, Zwahlen M, Thoma DS (2012). Systematic review of the survival rate and the incidence of biological, technical, and aesthetic complications of single crowns on implants reported in longitudinal studies with a mean follow-up of 5 years. Clin Oral Implants Res.

[10] Derks J, Tomasi C (2015). Peri-implant health and disease. A systematic review of current epidemiology. J Clin Periodontol.

[11] Esposito M, Hirsch JM, Lekholm U, Thomsen P (1998). Biological factors contributing to failures of osseointegrated oral implants. (II). Etiopathogenesis. Eur J Oral Sci.

[12] Lang NP, Wilson TG, Corbet EF (2000). Biological complications with dental implants: their prevention, diagnosis and treatment. Clin Oral Implants Res.

[13] Lang NP, Berglundh T (2011). Periimplant diseases: where are we now?--Consensus of the seventh european workshop on Periodontology. J Clin Periodontol.

[14] Mombelli A (1999). In vitro models of biological responses to implant microbiological models. Adv Dent Res.

[15] Karoussis IK, Müller S, Salvi GE, Heitz-Mayfield LJ, Brägger U, Lang NP (2004). Association between periodontal and peri-implant conditions: a 10-year prospective study. Clin Oral Implants Res.

[16] Van der Weijden GA, van Bemmel KM, Renvert S (2005). Implant therapy in partially edentulous, periodontally compromised patients: a review. J Clin Periodontol.

[17] Renvert S, Persson GR (2009). Periodontitis as a potential risk factor for peri-implantitis. J Clin Periodontol.

[18] Heitz-Mayfield LJ (2008). Peri-implant diseases: diagnosis and risk indicators. J Clin Periodontol.

[19] Heitz-Mayfield LJ, Huynh-Ba G (2009). History of treated periodontitis and smoking as risks for implant therapy. Int J Oral Maxillofac Implants.

[20] Sanz M, Chapple IL (2012). Clinical research on peri-implant diseases: consensus report of Working Group 4. J Clin Periodontol.

[21] Belibasakis GN, Charalampakis G, Bostanci N, Stadlinger B (2015). Peri-implant infections of oral biofilm etiology. Adv Exp Med Biol.

[22] Sousa V, Nibali L, Spratt D, Dopico J, Mardas N, Petrie A (2017). Peri-implant and periodontal microbiome diversity in aggressive periodontitis patients: a pilot study. Clin Oral Implants Res.

[23] Sahrmann P, Gilli F, Wiedemeier DB, Attin T, Schmidlin PR, Karygianni L. The Microbiome of Peri-Implantitis: a systematic review and Meta-analysis. Microorganisms. 2020;8(5). 10.3390/microorganisms8050661 PMC7284896 32369987

[24] Casado PL, Otazu IB, Balduino A, de Mello W, Barboza EP, Duarte ME (2011). Identification of periodontal pathogens in healthy periimplant sites. Implant Dent.

[25] Renvert S, Roos-Jansåker AM, Lindahl C, Renvert H, Rutger Persson G (2007). Infection at titanium implants with or without a clinical diagnosis of inflammation. Clin Oral Implants Res.

[26] Dabdoub SM, Tsigarida AA, Kumar PS (2013). Patient-specific analysis of periodontal and peri-implant microbiomes. J Dent Res.

[27] Koyanagi T, Sakamoto M, Takeuchi Y, Maruyama N, Ohkuma M, Izumi Y (2013). Comprehensive microbiological findings in peri-implantitis and periodontitis. J Clin Periodontol.

[28] Maruyama N, Maruyama F, Takeuchi Y, Aikawa C, Izumi Y, Nakagawa I (2014). Intraindividual variation in core microbiota in peri-implantitis and periodontitis. Sci Rep.

[29] Robitaille N, Reed DN, Walters JD, Kumar PS (2016). Periodontal and peri-implant diseases: identical or fraternal infections?. Mol Oral Microbiol.

[30] Daubert DM, Weinstein BF, Bordin S, Leroux BG, Flemming TF (2015). Prevalence and predictive factors for peri-implant disease and implant failure: a cross-sectional analysis. J Periodontol.

[31] Kumar PS, Mason MR, Brooker MR, O’Brien K (2012). Pyrosequencing reveals unique microbial signatures associated with healthy and failing dental implants. J Clin Periodontol.

[32] Mombelli A, Décaillet F (2011). The characteristics of biofilms in peri-implant disease. J Clin Periodontol.

[33] Persson GR, Renvert S (2014). Cluster of bacteria associated with peri-implantitis. Clin Implant Dent Relat Res.

[34] Yu XL, Chan Y, Zhuang L, Lai HC, Lang NP, Keung Leung W (2019). Intra-oral single-site comparisons of periodontal and peri-implant microbiota in health and disease. Clin Oral Implants Res.

[35] Sanz-Martin I, Doolittle-Hall J, Teles RP, Patel M, Belibasakis GN, Hämmerle CHF (2017). Exploring the microbiome of healthy and diseased peri-implant sites using Illumina sequencing. J Clin Periodontol.

[36] Ghensi P, Manghi P, Zolfo M, Armanini F, Pasolli E, Bolzan M (2020). Strong oral plaque microbiome signatures for dental implant diseases identified by strain-resolution metagenomics. NPJ Biofilms Microbiomes.

[37] Zhuang LF, Watt RM, Mattheos N, Si MS, Lai HC, Lang NP (2016). Periodontal and peri-implant microbiota in patients with healthy and inflamed periodontal and peri-implant tissues. Clin Oral Implants Res.

[38] Zheng H, Xu L, Wang Z, Li L, Zhang J, Zhang Q (2015). Subgingival microbiome in patients with healthy and ailing dental implants. Sci Rep.

[39] Tsigarida AA, Dabdoub SM, Nagaraja HN, Kumar PS (2015). The influence of smoking on the Peri-Implant Microbiome. J Dent Res.

[40] Tringe SG, von Mering C, Kobayashi A, Salamov AA, Chen K, Chang HW (2005). Comparative metagenomics of microbial communities. Science.

[41] Manichanh C, Rigottier-Gois L, Bonnaud E, Gloux K, Pelletier E, Frangeul L (2006). Reduced diversity of faecal microbiota in Crohn’s disease revealed by a metagenomic approach. Gut.

[42] Qin J, Li R, Raes J, Arumugam M, Burgdorf KS, Manichanh C (2010). A human gut microbial gene catalogue established by metagenomic sequencing. Nature.

[43] Cho-Yan Lee J, Mattheos N, Nixon KC, Ivanovski S (2012). Residual periodontal pockets are a risk indicator for peri-implantitis in patients treated for periodontitis. Clin Oral Implants Res.

[44] Glavind L, Löe H (1967). Errors in the clinical assessment of periodontal destruction. J Periodontal Res.

[45] Lang NP, Joss A, Orsanic T, Gusberti FA, Siegrist BE (1986). Bleeding on probing. A predictor for the progression of periodontal disease?. J Clin Periodontol.

[46] Renvert S, Persson GR, Pirih FQ, Camargo PM (2018). Peri-implant health, peri-implant mucositis, and peri-implantitis: case definitions and diagnostic considerations. J Periodontol.

[47] Tonetti MS, Greenwell H, Kornman KS (2018). Staging and grading of periodontitis: Framework and proposal of a new classification and case definition. J Periodontol.

[48] Martin M. Cutadapt removes adapter sequences from high-throughput sequencing reads. Embnet J. 2011;17(1).

[49] Chen S, Zhou Y, Chen Y, Gu J. Fastp: an ultra-fast all-in-one FASTQ preprocessor. Cold Spring Harbor Laboratory. 2018(17). 10.1093/bioinformatics/bty560 PMC6129281 30423086

[50] Wood DE, Lu J, Langmead B. Improved metagenomic analysis with Kraken 2. Genome Biol. 2019;20(1). 10.1186/s13059-019-1891-0 PMC6883579 31779668

[51] Caporaso J, Kuczynski J, Stombaugh J, Bittinger K, Bushman F. QIIME allows integration and analysis of high-throughput community sequencing data. Nat Meth. 2010. 10.1038/nmeth.f.303 PMC3156573 20383131

[52] Simpson EH (1997). Measurement of Diversity. J Cardiothorac Vasc Anesth.

[53] Ramette A (2007). Multivariate analyses in microbial ecology. FEMS Microbiol Ecol.

[54] Segata N, Izard J, Waldron L, Gevers D, Miropolsky L, Garrett WS (2011). Metagenomic biomarker discovery and explanation. Genome Biol.

[55] Kanehisa M, Goto S (2000). KEGG: kyoto encyclopedia of genes and genomes. Nucleic Acids Res.

[56] Yu C, Chen F, Jiang J, Zhang H, Zhou M (2019). Screening key genes and signaling pathways in colorectal cancer by integrated bioinformatics analysis. Mol Med Rep.

[57] Socransky SS, Haffajee AD, Cugini MA, Smith C, Kent RL (1998). Jr. Microbial complexes in subgingival plaque. J Clin Periodontol.

[58] Shiba T, Watanabe T, Kachi H, Koyanagi T, Maruyama N, Murase K (2016). Distinct interacting core taxa in co-occurrence networks enable discrimination of polymicrobial oral diseases with similar symptoms. Sci Rep.

[59] Komatsu K, Shiba T, Takeuchi Y, Watanabe T, Koyanagi T, Nemoto T (2020). Discriminating Microbial Community structure between Peri-Implantitis and Periodontitis with Integrated Metagenomic, Metatranscriptomic, and Network Analysis. Front Cell Infect Microbiol.

[60] Charalampakis G, Belibasakis GN (2015). Microbiome of peri-implant infections: lessons from conventional, molecular and metagenomic analyses. Virulence.

[61] Shibli JA, Melo L, Ferrari DS, Figueiredo LC, Faveri M, Feres M (2008). Composition of supra- and subgingival biofilm of subjects with healthy and diseased implants. Clin Oral Implants Res.

[62] Mombelli A, Marxer M, Gaberthüel T, Grunder U, Lang NP (1995). The microbiota of osseointegrated implants in patients with a history of periodontal disease. J Clin Periodontol.

[63] Takanashi K, Kishi M, Okuda K, Ishihara K (2004). Colonization by Porphyromonas gingivalis and Prevotella intermedia from teeth to osseointegrated implant regions. Bull Tokyo Dent Coll.

[64] Tabanella G, Nowzari H, Slots J (2009). Clinical and microbiological determinants of ailing dental implants. Clin Implant Dent Relat Res.

[65] Shchipkova AY, Nagaraja HN, Kumar PS (2010). Subgingival microbial profiles of smokers with periodontitis. J Dent Res.

[66] Koyanagi T, Sakamoto M, Takeuchi Y, Ohkuma M, Izumi Y. Analysis of microbiota associated with peri-implantitis using 16S rRNA gene clone library. J Oral Microbiol. 2010;2. 10.3402/jom.v2i0.5104 PMC3084566 21523229

[67] Pérez-Chaparro PJ, Gonçalves C, Figueiredo LC, Faveri M, Lobão E, Tamashiro N (2014). Newly identified pathogens associated with periodontitis: a systematic review. J Dent Res.

[68] Papapanou PN, Park H, Cheng B, Kokaras A, Paster B, Burkett S (2020). Subgingival microbiome and clinical periodontal status in an elderly cohort: the WHICAP ancillary study of oral health. J Periodontol.

[69] Eick S, Ramseier CA, Rothenberger K, Brägger U, Buser D, Salvi GE (2016). Microbiota at teeth and implants in partially edentulous patients. A 10-year retrospective study. Clin Oral Implants Res.

[70] Colombo AP, Bennet S, Cotton SL, Goodson JM, Kent R, Haffajee AD (2012). Impact of periodontal therapy on the subgingival microbiota of severe periodontitis: comparison between good responders and individuals with refractory periodontitis using the human oral microbe identification microarray. J Periodontol.

[71] Yu Y, Qiu L, Guo J, Yang D, Qu L, Yu J (2015). TRIB3 mediates the expression of Wnt5a and activation of nuclear factor-κB in Porphyromonas endodontalis lipopolysaccharide-treated osteoblasts. Mol Oral Microbiol.

[72] Marchesan J, Jiao Y, Schaff RA, Hao J, Morelli T, Kinney JS (2016). TLR4, NOD1 and NOD2 mediate immune recognition of putative newly identified periodontal pathogens. Mol Oral Microbiol.

[73] Apatzidou D, Lappin DF, Hamilton G, Papadopoulos CA, Konstantinidis A, Riggio MP (2017). Microbiome of peri-implantitis affected and healthy dental sites in patients with a history of chronic periodontitis. Arch Oral Biol.

[74] Abusleme L, Dupuy AK, Dutzan N, Silva N, Burleson JA, Strausbaugh LD (2013). The subgingival microbiome in health and periodontitis and its relationship with community biomass and inflammation. Isme j.

[75] Kistler JO, Booth V, Bradshaw DJ, Wade WG (2013). Bacterial community development in experimental gingivitis. PLoS ONE.

[76] Deng ZL, Sztajer H, Jarek M, Bhuju S, Wagner-Döbler I (2018). Worlds apart - transcriptome profiles of key oral microbes in the Periodontal Pocket compared to single Laboratory Culture reflect synergistic interactions. Front Microbiol.

[77] Meffert RM (1996). Periodontitis vs. peri-implantitis: the same disease? The same treatment?. Crit Rev Oral Biol Med.

[78] Zhang Q, Qin XY, Jiang WP, Zheng H, Xu XL, Chen F (2015). Comparison of Subgingival and Peri-implant Microbiome in Chronic Periodontitis. Chin J Dent Res.

[79] Murugkar PP, Collins AJ, Chen T, Dewhirst FE (2020). Isolation and cultivation of candidate phyla radiation Saccharibacteria (TM7) bacteria in coculture with bacterial hosts. J Oral Microbiol.

[80] Cross KL, Campbell JH, Balachandran M, Campbell AG, Cooper CJ, Griffen A (2019). Targeted isolation and cultivation of uncultivated bacteria by reverse genomics. Nat Biotechnol.

[81] Chipashvili O, Utter DR, Bedree JK, Ma Y, Schulte F, Mascarin G (2021). Episymbiotic Saccharibacteria suppresses gingival inflammation and bone loss in mice through host bacterial modulation. Cell Host Microbe.

[82] Bolstad AI, Jensen HB, Bakken V (1996). Taxonomy, biology, and periodontal aspects of Fusobacterium nucleatum. Clin Microbiol Rev.

[83] Teles R, Sakellari D, Teles F, Konstantinidis A, Kent R, Socransky S (2010). Relationships among gingival crevicular fluid biomarkers, clinical parameters of periodontal disease, and the subgingival microbiota. J Periodontol.

[84] Heydenrijk K, Raghoebar GM, Meijer HJ, van der Reijden WA, van Winkelhoff AJ, Stegenga B (2002). Two-stage IMZ implants and ITI implants inserted in a single-stage procedure. A prospective comparative study. Clin Oral Implants Res.

[85] Lindhe J, Meyle J (2008). Peri-implant diseases: Consensus Report of the Sixth European Workshop on Periodontology. J Clin Periodontol.

[86] Hajishengallis G, Lamont RJ (2016). Dancing with the Stars: how choreographed bacterial interactions dictate nososymbiocity and give rise to keystone pathogens, accessory pathogens, and Pathobionts. Trends Microbiol.

[87] Elhadad D, Desai P, Rahav G, McClelland M, Gal-Mor O (2015). Flagellin is required for host cell Invasion and Normal Salmonella Pathogenicity Island 1 expression by Salmonella enterica Serovar Paratyphi A. Infect Immun.

[88] Ohnishi K, Ohto Y, Aizawa S, Macnab RM, Iino T (1994). FlgD is a scaffolding protein needed for flagellar hook assembly in Salmonella typhimurium. J Bacteriol.

[89] Deng ZL, Szafrański SP, Jarek M, Bhuju S, Wagner-Döbler I (2017). Dysbiosis in chronic periodontitis: key microbial players and interactions with the human host. Sci Rep.

[90] Dashper SG, Seers CA, Tan KH, Reynolds EC (2011). Virulence factors of the oral spirochete Treponema denticola. J Dent Res.

[91] Charon NW, Goldstein SF (2002). Genetics of motility and chemotaxis of a fascinating group of bacteria: the spirochetes. Annu Rev Genet.

[92] Shi B, Chang M, Martin J, Mitreva M, Lux R, Klokkevold P (2015). Dynamic changes in the subgingival microbiome and their potential for diagnosis and prognosis of periodontitis. mBio.

[93] Hidaka T, Mori M, Imai S, Hara O, Nagaoka K, Seto H (1989). Studies on the biosynthesis of bialaphos (SF-1293). 9. Biochemical mechanism of C-P bond formation in bialaphos: discovery of phosphoenolpyruvate phosphomutase which catalyzes the formation of phosphonopyruvate from phosphoenolpyruvate. J Antibiot (Tokyo).

[94] Lux R, Shi W (2004). Chemotaxis-guided movements in bacteria. Crit Rev Oral Biol Med.

[95] Abiko Y, Sato T, Mayanagi G, Takahashi N (2010). Profiling of subgingival plaque biofilm microflora from periodontally healthy subjects and from subjects with periodontitis using quantitative real-time PCR. J Periodontal Res.

[96] Tamura N, Ochi M, Miyakawa H, Nakazawa F (2013). Analysis of bacterial flora associated with peri-implantitis using obligate anaerobic culture technique and 16S rDNA gene sequence. Int J Oral Maxillofac Implants.

[97] Hajishengallis G, Lambris JD (2012). Complement and dysbiosis in periodontal disease. Immunobiology.

[98] Bubier JA, Chesler EJ, Weinstock GM (2021). Host genetic control of gut microbiome composition. Mamm Genome.

[99] Demmitt BA, Corley RP, Huibregtse BM, Keller MC, Hewitt JK, McQueen MB (2017). Genetic influences on the human oral microbiome. BMC Genomics.

[100] Yan L, Ma C, Wang D, Hu Q, Qin M, Conroy JM (2012). OSAT: a tool for sample-to-batch allocations in genomics experiments. BMC Genomics.

[101] Laurin-Lemay S, Brinkmann H, Philippe H (2012). Origin of land plants revisited in the light of sequence contamination and missing data. Curr Biol.

[102] Eisenhofer R, Minich JJ, Marotz C, Cooper A, Knight R, Weyrich LS (2019). Contamination in low microbial biomass Microbiome Studies: issues and recommendations. Trends Microbiol.

[103] Hänfling B, Lawson Handley L, Read DS, Hahn C, Li J, Nichols P (2016). Environmental DNA metabarcoding of lake fish communities reflects long-term data from established survey methods. Mol Ecol.

[104] Goldberg CS, Turner CR, Deiner K, Klymus KE, Thomsen PF, Murphy MA (2016). Critical considerations for the application of environmental DNA methods to detect aquatic species. Methods Ecol Evol.

[105] Glassing A, Dowd SE, Galandiuk S, Davis B, Chiodini RJ (2016). Inherent bacterial DNA contamination of extraction and sequencing reagents may affect interpretation of microbiota in low bacterial biomass samples. Gut Pathog.

