# Supplementary material for: Metagenomic analysis of healthy and diseased peri-implant microbiome under different periodontal conditions: a cross-sectional study
Source: BMC Oral Health. 2024 Jan 17;24:105. doi: 10.1186/s12903-023-03442-9 (PMC10795403; doi:10.1186/s12903-023-03442-9)
Supplement: Supplementary file 1 — Supplementary Material 1 [file 12903_2023_3442_MOESM1_ESM.docx]

**Supplementary Information 1**


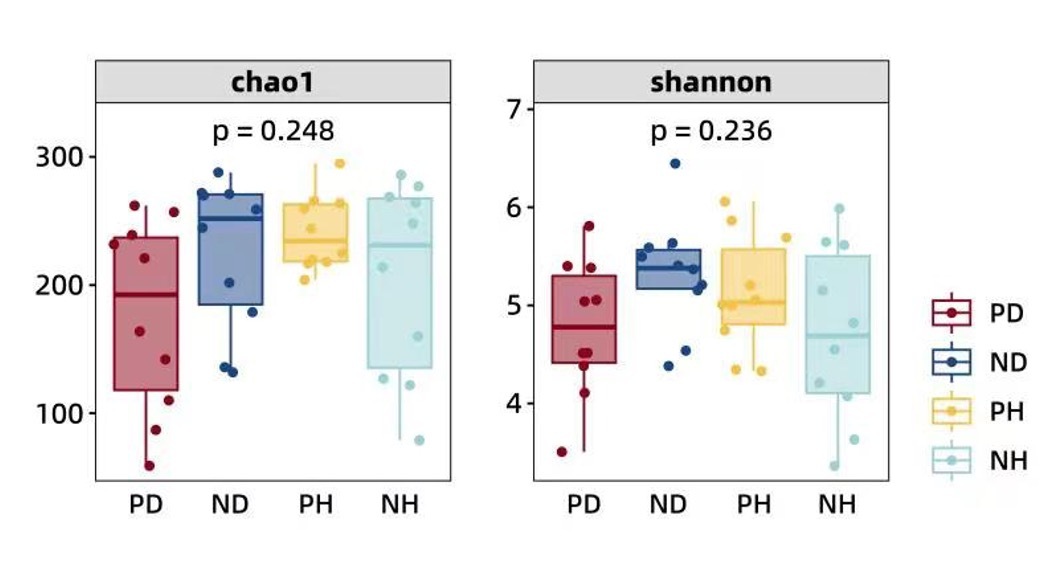


**Additional Fig. 1** Alpha-diversity distributions shows lower richness and evenness in PD group comparing to ND group. *p* values were obtained by the Kruskal-Wallis rank sum test.


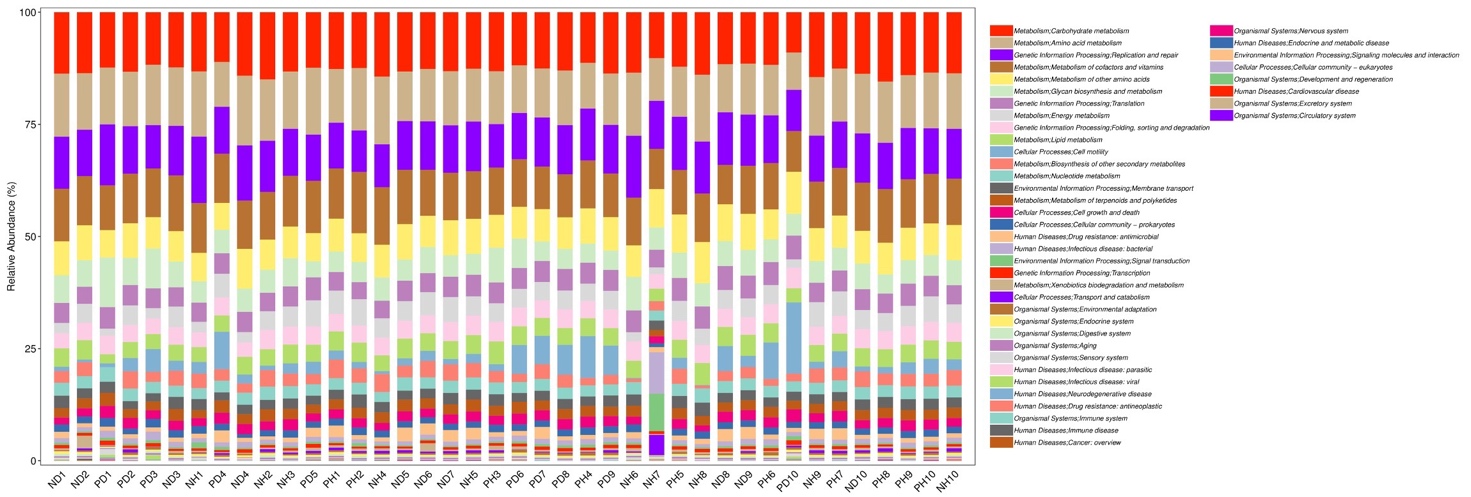


**Additional Fig. 2** Overall functional composition of each subject from four study groups at KEGG level 2 shows similar predominant function units across groups.
